# A Rapid LC-HRMS Method for the Determination of Domoic Acid in Urine Using a Self-Assembly Pipette Tip Solid-Phase Extraction

**DOI:** 10.3390/toxins8010010

**Published:** 2015-12-29

**Authors:** Yiping Zhang, Dawei Chen, Zhuan Hong

**Affiliations:** 1 Third Institute of Oceanography State Oceanic Administration, Xiamen 361005, China; ypzhang@tio.org.cn; 2 Fujian Collaborative Innovation Center for Exploitation and Utilization of Marine Biological Resources, Xiamen 361005, China; 3 Key Laboratory of Food Safety Risk Assessment, Ministry of Health, China National Center for Food Safety Risk Assessment, Beijing 100021, China

**Keywords:** domoic acid, urine, pipette tip solid-phase extraction, amnesic shellfish poisoning, high resolution mass spectrometry

## Abstract

In this study, we developed a self-assembly pipette tip solid-phase extraction (PTSPE) method using a high molecular weight polymer material (PAX) as the adsorbent for the determination of domoic acid (DA) in human urine samples by liquid chromatography high-resolution mass spectrometry (LC-HRMS) analysis. The PTSPE cartridge, assembled by packing 9.1 mg of PAX as sorbent into a 200 μL pipette tip, showed high adsorption capacity for DA owing to the strong cationic properties of PAX. Compared with conventional SPE, the PTSPE is simple and fast, and shows some advantages in the aspects of less solvent consumption, low cost, the absence of the evaporation step, and short time requirement. All the parameters influencing the extraction efficiency such as pH, the amount of sorbent, the number of aspirating/dispensing cycles, and the type and volume of eluent in PTSPE were carefully investigated and optimized. Under the optimized conditions, the limit of detection (LOD) and limit of quantification (LOQ) values of DA were 0.12 μg/L and 0.37 μg/L respectively. The extraction recoveries of DA from the urine samples spiked at four different concentrations were in a range from 88.4% to 102.5%. The intra- and inter-day precisions varied from 2.1% to 7.6% and from 2.6% to 12.7%, respectively. The accuracy ranged from −1.9% to −7.4%.

## 1. Introduction

**Figure 1:**
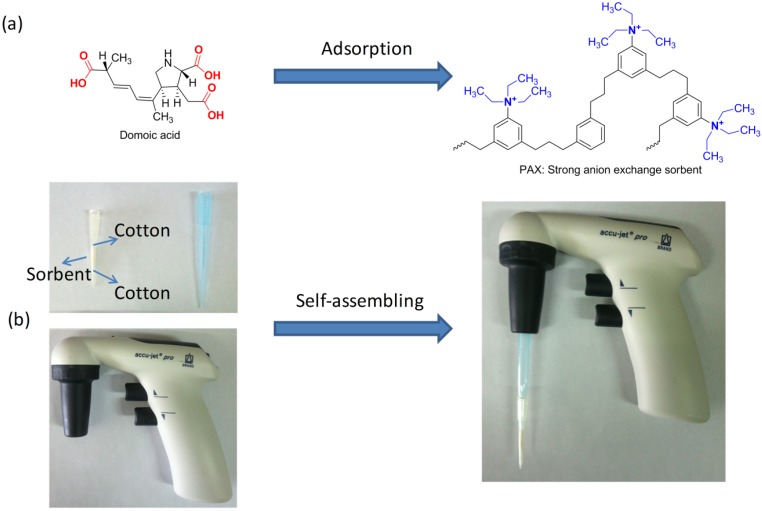
(**a**) Molecular structure of DA and schematic of PAX; and (**b**) self-assembly pipette tip solid-phase extraction procedure.

Domoic acid (DA, Figure 1a), a major amnesic shellfish poisoning (ASP) toxin, is a naturally-occurring hydrophilic neurotoxic amino acid produced by algae (e.g., *Chondria armata*) and accumulates in shellfish, sardines, and anchovies [1]. Consumption of DA-contaminated shellfish is responsible for severe neurological symptom disorders, such as headache, disorientation, confusion, and short-term memory loss [2,3]. The neurological effects of DA have been attributed to several mechanisms, but the one of concern is through glutamate receptors. As an excitatory amino acid analogue of glutamate, DA displays a very strong affinity for the glutamate receptors, which results in excitotoxicity initiated by an integrative action on ionotropic glutamate receptors. In addition, DA binds predominately to *N*-methyl-D-aspartate (NMDA) receptors in the central nervous system [4,5,6]. Therefore, DA is considered a significant risk to public health and to the economic viability of the aquaculture and shellfish harvesting industries. To ensure human health protection, monitoring programs are carried out in many countries and several analytical methods have been developed for the monitoring of DA in shellfish, phytoplankton, and seawater [7,8,9,10,11,12,13]. The European Community’s regulations have required the concentration of DA in shellfish should not exceed the permissible limit of 20 mg/kg [14]. However, only a few studies have been reported for the determination of DA in body fluids using sensitive analytical methods [15,16]. In suspected cases of DA poisoning, the gastrointestinal tract of animals is usually completely empty because of the direct emetic effect of DA, but serum and urine are easily available in such cases. Moreover, kinetic studies in monkeys and rats have demonstrated that DA is primarily excreted unchanged in urine following absorption [17,18,19]. The concentrations of DA levels in urine samples of marine mammals have been reported to be in the range 5–10,000 µg/L, whereas serum levels ranged from 5–180 µg/L [20]. Additionally, urine is the most useful bio sample in poisoning cases because determinations of DA up to 72 h are possible, whereas for plasma or serum analysis times are limited to within 30 h [21,22].

In the past 20 years, liquid chromatography-mass spectrometry (LC-MS) has become the most commonly-used analytical technique in clinical bioanalysis. Nevertheless, it is necessary to perform sample pretreatment of urine before LC-MS analysis because of the complexity inherent to urine (e.g., salts and moderate-to-high levels of proteins) [23]. Solid-phase extraction (SPE) is a common method that is usually extensively applied in sample preparation before the quantitative analysis of the target analytes. The commercially available SPE phases used for the cleanup in DA assay include reversed phase C_18_ [24], strong or weak anion exchange [25,26] and strong cation-exchange cartridges [27]. In addition, some references have reported using sol-gel amorphous titania (TiO_2_), molecularly-imprinted polymer as the solid phase sorbents for the extraction of DA from several matrices [28,29]. Although the SPE method had been verified to be effective for cleaning up the complex matrices and achieving high pre-concentration factors, the approach is relatively expensive and time-consuming. Recently, a miniature model of SPE, the pipette tip solid-phase extraction (PTSPE) method has been reported and applied in bioanalysis based on the use of micro amounts of the sorbent phase [30,31,32]. The PTSPE method exhibits some advantages over the traditional SPE method including lower solvent consumption, lower cost, the absence of an evaporation step and shorter time requirement.

In this study, we developed a self-assembly PTSPE method using a high molecular weight polymer anion exchange material (PAX) as the adsorbent for the determination of DA in urine by liquid chromatography high-resolution mass spectrometry (LC-HRMS) analysis. The assembly of the PTSPE procedure was simple, easy, and inexpensive (Figure 1b). PTSPE was performed in a semi-automated pipette controller to reduce the overall extraction time. In addition, key parameters that affected the extraction efficiency in the PTSPE procedure, such as the selection and amount of sorbent, the pH of the extract, the number of aspirating/dispensing cycles, and the type and volume of eluent were carefully investigated and optimized. In chemical analysis, optimization of each experimental variable is desirable to ensure the best possible analytical response [33]. Recently, the multivariate designs of experiments (DOE) has been widely employed to obtain the optimum experimental parameters [34,35]. The experimental design employed in this study has taken advantage of a reduced number of experiments and results in less time, effort and use of resources than the one-factor analysis approach. More importantly, the interactions between variables and the non-linear relationships of the analytical responses can be taken into account by the DOE approach. A central composite design (CCD) was presented by Box and Wilson [36] and became one of the most frequent designs used to fit quadratic models. A CCD combines a two-level factorial design with axial points and at least one point at the center of the experimental region to fit quadratic polynomial. The central points are usually repeated to obtain a good estimation of experimental error. Based on these criteria, a CCD approach was used for studying the optimum variables in this PTSPE procedure after undertaking a preliminary one-factor analysis of variables.

## 2. Results and Discussion

### 2.1. Separation of Domoic Acid

**Figure 2:**
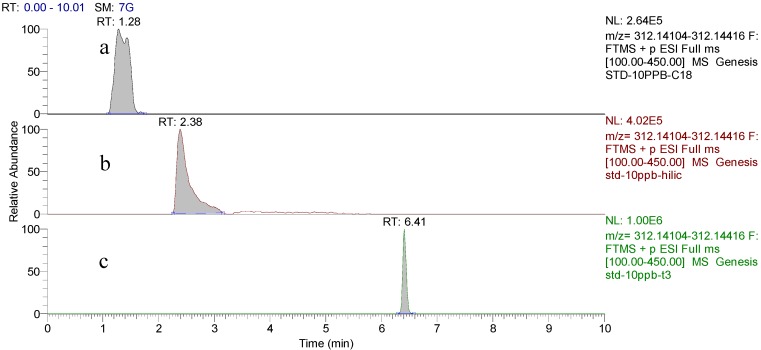
Chromatograms for DA at a concentration of 10 μg/L acquired with different columns: (**a**) BEH C_18_; and (**b**) BEH HILIC; (**c**) HSS T_3_.

The effects of different LC columns on analyte separation and retention were initially studied. Three different types of analytical columns including a Waters BEH C_18_ (2.1 mm × 100 mm, 1.7 μm), a Waters BEH HILIC (2.1 mm × 100 mm, 1.7 μm), and a Waters HSS T_3_ (2.1 mm × 100 mm, 1.8 μm) were tested under their optimal elution conditions, respectively, which were shown in the Supplementary Data. The results indicated that DA had superior retention and symmetrical peak with favorable MS response in T_3_ column (Figure 2). As for the mobile phase, acetonitrile and water with a variety of modifiers were investigated. It was found that a mixture of 4 mM ammonium formate and 0.1% formic acid solution gave acceptable performance in terms of peak shape and ionization of DA under the optimized linear gradient mode (Figure S1, Supplementary Data).

### 2.2. HR-MS/MS Analysis

**Figure 3:**
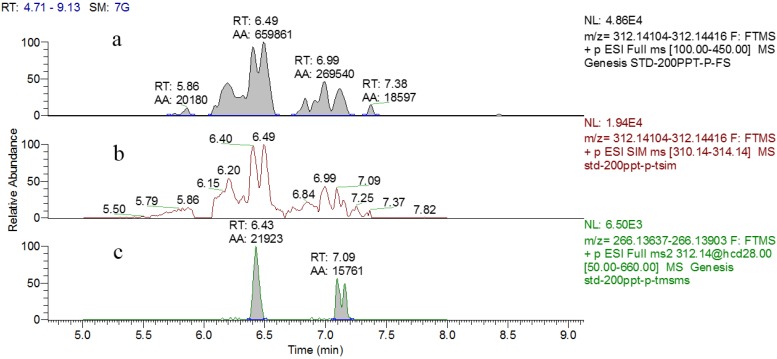
Chromatographs for DA at the concentration of 0.2 μg/L in a blank urine extract for the acquisition modes: (**a**) full scan; (**b**) tSIM; and (**c**) tMSMS.

Full scan, targeted, single-ion monitoring (tSIM) and targeted MS/MS (tMS/MS) modes are three kinds of commonly used quantitative models for the Q Exactive mass spectrometer and all were evaluated and optimized in the present study. Full scan mode is a full scan of all ions in the specified mass range. In the tSIM mode, the precursor in the inclusion list (with retention times) can be selected in the quadrupole with a particular isolation width. In the tMS/MS mode, the precursor specified in the inclusion list is selected by the quadrupole, and fragmented in a high energy collisional dissociation (HCD) cell with specific fragmentation energy. The results for DA (0.2 μg/L spiked in blank urine) for these three acquisition modes within individual conditions are shown in Figure 3. This figure highlights the fact that tMS/MS is more selective and specific than the tSIM and full scan modes as a consequence of the complexity of the urine matrices, which leads to better limit of detection. Therefore, the tMS/MS mode was selected for the determination of DA in urine. Using tMS/MS mode, the parent ion was selected in the quadruple (312.14 *m*/*z* for DA) using a specified *m*/*z* window (4 Da) and subsequently fragmented in the HCD cell. As shown in Figure S2, Supplementary Data, a full scan of all fragmented ions (e.g., 312.1429, 266.1376, 220.1323, 161.0955 *m*/*z* for DA) originating from the parent ion was performed, and 266.1376 *m*/*z* was used for quantitative analysis. After selection of the product ions, fragmentation energy scans were carried out to obtain the optimal NCE (28%) for the complete fragmentation of precursor ions.

### 2.3. Optimization of PTSPE Procedure by the One-Factor Analysis of Variables

Analyte recovery is a good measure of the extraction efficiency for DA in the PTSPE procedure [37]. To obtain high recoveries, experimental parameters, including the type of sorbent, the pH, the amount of sorbent, the number of aspirating/dispensing cycles, the type and the volume of eluent were optimized based on the results for analyses of spiked urine samples containing 1 μg/L DA. The following procedure was used to optimize the PTSPE method, and the specific extraction conditions for optimization of each parameter are shown in Table S1, Supplementary Data.

**Figure 4:**
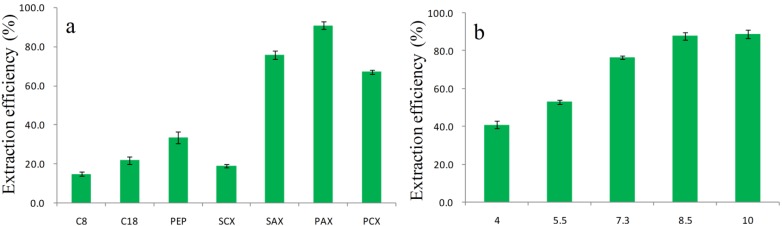
Effects of the different sorbents (**a**) and pH (**b**) on the extraction efficiency (*n* = 3) of DA in urine.

Selection of an effective sorbent is clearly important for the PTSPE procedure [30,31,32]. The urine samples spiked with DA were extracted using 10 mg C_18_, C_8_, PEP, SCX, SAX, PAX, and PCX as sorbents respectively, in our preliminary studies (Figure 4a). It was found that the best adsorption efficiency was obtained with PAX. As shown in Figure 1a, DA is characterized by the presence of three carboxyl groups and one amino group with pKa values of 1.85, 4.47, 4.75, and 10.60, respectively. Therefore, DA exists as an anionic species with different charge states over a wide pH range, and this species is more readily adsorbed by the SAX and PAX sorbents (based on the characteristics of strong cationic structure) than the SCX and PCX sorbents (based on the characteristics of strong anionic structure); however, because of the lower surface area of SAX, its adsorption capacity was still inferior to that of PAX. For the other sorbents, C_8_, C_18_, and PEP (all reversed phase sorbents) yielded low adsorption efficiencies, which indicated that a 10 mg sorbent loading was insufficient to retain DA owing to its high polarity. Therefore, based on these results, PAX was selected as a suitable sorbent for further investigation.

Sample pH is one of the key parameters that control the extraction efficiency of acidic or basic components [38]. Generally, pH values affect extraction efficiency by altering the charge on the analyte or sorbent. DA, which has three carboxyl groups, is readily adsorbed by PAX as an anionic species when the solution is alkaline. Thus, the effects on recovery at different pH values (4–10) and the lack of pH adjustment (pH = 7.3) were investigated (Figure 4b). There were no noticeable differences in the recoveries of DA in alkaline solution. In contrast analyte recovery was relatively low in acidified solutions (53% lower) and in the absence of pH adjustment (76% lower). Therefore, 100 μL of 2% ammonium hydroxide was added to 100 μL urine sample to maintain alkaline conditions.

**Figure 5:**
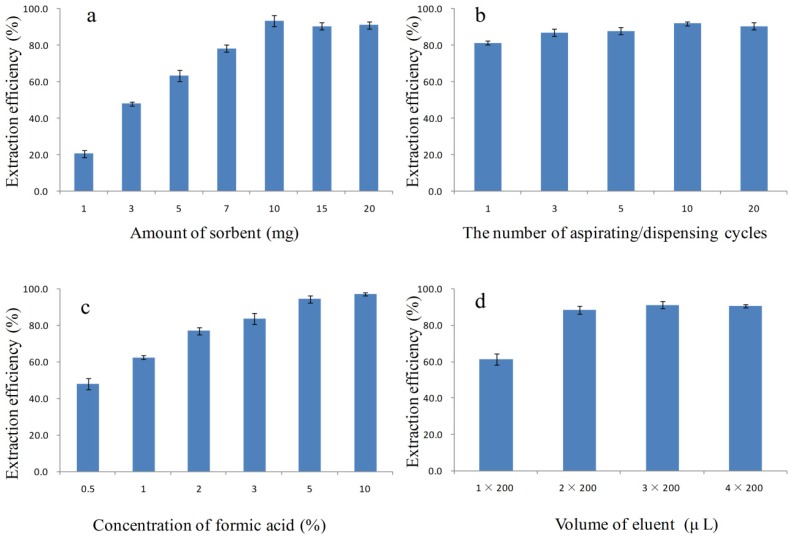
Effects of the PTSPE procedure conditions for recovery of DA (*n* = 3): (**a**) the amount of sorbent; (**b**) the number of aspirationg/dispensing cycles; (**c**) the concentration of formic acid and (**d**) the volume of eluent.

The effects of the amount of PAX sorbent (1–15 mg) packed into the pipette tip were investigated. As shown in Figure 5a, the adsorption rates were higher as the amount of PAX sorbent increased from 1–10 mg for DA. However, when the amount of PAX sorbent was greater than 10 mg, a further increase in the adsorption rate did not occur, which indicated that 10 mg of packing amount was sufficient to retain DA.

The number of aspirating/dispensing cycles is a critical parameter for achieving good extraction recovery of analyte by the PTSPE. In this study, the extraction of DA obtained equilibrium after three aspirating/dispensing cycles (Figure 5b) with an extraction time of approximately 10 s being used for one aspirating/dispensing cycle. The results showed that the adsorption of DA onto PAX sorbent was a fast process with fewer aspirating/dispensing cycles and less extraction time than that for other sorbents, such as C_18_. As a result, three aspirating/dispensing cycles were used in subsequent experiments.

The type and the volume of desorption solvent are critical for sample purification efficiency and achieving high analyte recovery. It is well known that acid substances are easily eluted from a strong anion exchange sorbent in acid conditions [39]. Moreover, it is desirable to use a mobile phase in the sample preparation process as this facilitates direct injection into the LC system [40]. Considering the solvent effect for DA in T3 column (Figure S3, Supplementary Data), various concentrations of formic acid solution were used for elution of DA from the PAX sorbent. As shown in Figure 5c, as the concentration of formic acid increased, higher extraction efficiency was found. However, no additional enhancement in extraction efficiency was observed for formic acid concentration >5%. In the following study, the volume of eluent needed to obtain optimal elution of DA was studied by undertaking multiple loadings (up to five times) of the small sample volumes (200 μL) of eluent. As indicated in Figure 5d, the recovery values of DA did not increase significantly for more than two eluent loadings. Therefore, the 2 × 200 μL eluent solution was considered sufficient for the complete elution of the analyte.

### 2.4. Design of Experiments

**Figure 6:**
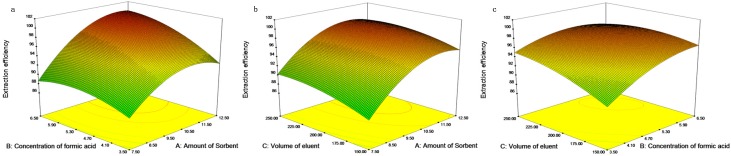
Three-dimensional plots of the effects of the amount of sorbent (A), the concentration of formic acid (B), and the volume of eluent (C) on efficiency of extraction of DA: (**a**) fixed C = 2 × 200 μL; (**b**) fixed B = 5%; (**c**) fixed A = 10 mg.

In initial studies concerning one-factor analysis of variables, it was found that the amount of sorbent, the concentration of formic acid and the volume of eluent were three important variables, which had a significant positive effect on the extraction efficiency. In order to ascertain the optimum PTSPE operating procedure and take into account interactions between variables, a CCD approach was carried out based on the preliminary experimental results obtained by the one-factor analysis of variables. The software Design-Expert (version 8.0.5b, Stat-Ease, Inc., Minneapolis, MN, USA) was used for experimental design, data analysis, and model-building for response surface methodology (RSM). The CCD requires an experiment number according to *N* = 2*^k^* + 2*k* + *c*_p_, where k is the factor number and *c*_p_ is the replicate number of the central point [33]. The effects of the amount of sorbent (7.5–12.5 mg, A), the concentration of formic acid (3.5%–6.5%, B) and the volume of eluent with two loadings (2 × 150 μL–2 × 250 μL, C) on the efficiency of extraction for the analyte were considered and optimized in CCD. A five-level, three-parameter experimental design of CCD approach was performed in random order for 20 run experiments, as outlined in Table 1. The full CCD was described as follows: (1) a full two-level (−1 and +1) factorial design; (2) a center point (*c*_p_ = 5); and (3) two axial points on the axis of each design variable at a distance of α = 2.0 from the design center. After performing the 20 run experiments (Table S2, Supplementary Data), the response *Y* was calculated based on the efficiency of extraction for DA, and all statistical analyses were performed by Design-Expert software. The ANOVA analysis for the response surface quadratic model showed that the model was significant with an *F*-value of 6.57 and a *p*-value of 0.0035 being obtained. The lack of fit of the model relative to its pure error showed an *F*-value of 2.65 and a *p*-value of 0.1542, which indicated that the fitted model was considered adequate to predict the efficiency of extraction under any combinations of the experimental variables. Model coefficients for the response were shown in Table S3, Supplementary Data. The final equation in terms of coded factors was: Response = 97.34 + 4.12 A + 2.25 B + 1.50 C + 1.50 AB − 0.25 AC − 1.25 BC − 3.58 A^2^ − 1.70 B^2^ − 1.08 C^2^. The RSM was used to determine the optimum response for DA extraction. Three-dimensional (3D) surface response plots and their related counters, obtained using the fitted model, were shown in Figure 6. Figure 6 indicated that the estimated response surfaces, obtained from the quadratic model that exhibited the effects and interaction of two independent variables on the response as the third independent variable, was fixed at the central experimental level of zero. The results showed that the concentration of formic acid and the amount of sorbent were two key variables which had a significant positive effect on the extraction efficiency. However, the volume of eluent did not produce a significant effect. The optimum conditions were selected based on the analysis data obtained from the response surface plots and the regression coefficient plots. The experimental settings for the variables that maximize the efficiency of extraction (>95%) for DA were chosen in response optimization. Among these settings, the most desirable variable levels ranged as follows: 9.1–12.5 mg for the amount of sorbent, 4.0%–6.5% for the concentration of formic acid, and 2 × 150 μL–2 × 250 μL for the volume of eluent. In order to obtain the maximum response together with minimal amount of sorbent consumption, the best combination was found to be 9.1 mg PAX sorbent and 2 × 185 μL of 6% formic acid solution. Under such conditions, analyte recovery was 95.3%.

**Table 1:** Variables and levels evaluated in the central composite design.

Independent variables	Unit	Symbol	Coded Level				
			−α	−1	0	+1	+α
Amount of sorbent	mg	A	5	7.5	10	12.5	15
Concentration of formic acid	%	B	2	3.5	5	6.5	8
Volume of eluent	μL	C	2 × 100	2 × 150	2 × 200	2 × 250	2 × 300

For all optimized conditions, the pretreatment procedure by the PTSPE method was simple and fast. Furthermore, the cost of PAX sorbent for one sample is lower (approximately 0.10 US dollars) compared with that by SPE column (at least $2.00 USD for each). In addition, the total time required for the extraction and cleanup of one sample was approximately 5 min.

### 2.5. Method Validation

**Figure 7:**
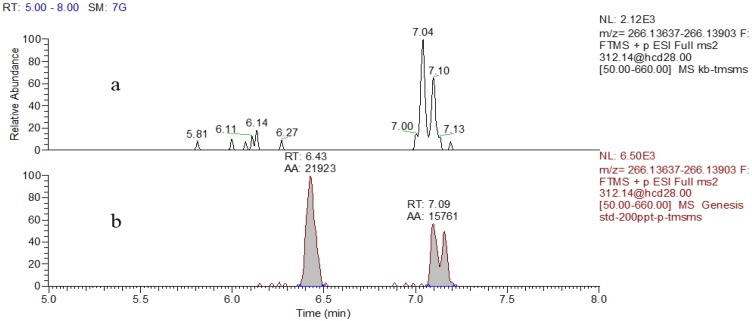
Extracted ion chromatograms for DA in the blank urine (**a**) and standard at 0.2 μg/L (**b**).

Given the high resolving power and accurate mass measurements of high-resolution mass spectrometry, the proposed method was considered to offer high specificity. This was confirmed by the absence of interfering peaks in the blank urine as noted in the tMSMS scan at the retention time for DA. Representative chromatographs including those of a blank urine and a DA standard (0.2 μg/L) are showed in Figure 7.

The calibration curve was linear over the validated concentration range (0.1, 0.2, 0.5, 1, 2, 5, and 10 μg/L). The linear regression equation for the calibration curve and *r*-value were: *y* = 44023.1*x* + 196.514, r = 0.9999, where *x* was the concentration of DA and y was the peak area of DA. The LOD value for DA was 0.12 μg/L, and the LOQ value was 0.37 μg/L when 100 μL urine was analyzed which were sufficiently low to meet the requirements of routine monitoring and exposure assessment.

The precision and accuracy data for QC samples (*n* = 5) at four levels of are presented in Table 2. The intra- and inter-day precision ranged from 2.1% to 7.6% (RSD_r_) and from 2.6% to 12.7% (RSD_R_), respectively. The intra- and inter-day accuracy ranged from −1.9% to −6.5% and from −3.1% to −7.4%, respectively. The results, which were within the acceptable criteria for accuracy and precision, allowed the accurate assay of the analyte in urine.

**Table 2:** Precision, accuracy, and recovery for DA.

Norminal Concentration (μg/L)	Intra-Day		Inter-Day		Analyte Recovery (%, *n* = 6)	Matrix Effect (%, *n* = 3)
	RSD_r_ (%)	Biases (%)	RSD_R_ (%)	Biases (%)		
0.37	7.6	−6.5	10.4	−7.4	88.4 ± 8.9	96.8 ± 2.1
0.74	5.8	−4.7	12.7	−5.3	92.8 ± 5.3	97.7 ± 2.6
3.7	2.1	−1.9	3.6	−3.1	97.2 ± 2.7	100.9 ± 1.4
37	2.4	−3.1	2.6	−4.2	102.5 ± 4.3	101.7 ± 1.2

The analyte recoveries for the QC samples are presented in Table 2. The recovery values ranged from 88.4% to 102.5%, which indicated that the overall recovery of DA was efficient and reproducible. Evaluation of matrix effect on the quantitative analysis of biological fluids is a further consideration in bioanalytical method validation. Table 2 shows the results of matrix effect for DA indicating that no significant matrix effects for DA were observed at each QC level. The variation (%RSD) was between 1.2% and 2.6%.

The results of the stability test are shown in Table 3. In all stability tests, the recoveries (84.2%–99.5%) for DA were acceptable with precision values ranging from 1.3% to 11.3%.

**Table 3:** Stability data for DA in urine under various conditions (*n* = 3).

Norminal Concentration (μg/L)	One Week		Two Weeks		Four Weeks	
	Recovery (%)	Precision (%)	Recovery (%)	Precision (%)	Recovery (%)	Precision (%)
0.37	88.1	8.7	84.2	9.2	87.2	10.5
0.74	89.4	10.7	90.6	11.3	91.1	7.9
3.7	98.4	3.8	97.9	3.4	99.2	2.4
37	99.5	1.8	96.2	4.1	95.6	1.3

## 3. Experimental Section

### 3.1. Chemicals and Reagents

Acetonitrile and methanol (HPLC grade) were obtained from Fisher Scientific (Fair Lawn, NJ, USA). Ammonium hydroxide, formic acid, and ammonium formate (HPLC grade) were purchased from Tedia (Weston, FL, USA). Ultra-pure water was prepared using a Milli-Q Plus system at 18.2 MΏ (Millipore, Bedford, MA, USA). C_18_, C_8_, PEP, SCX, SAX, PAX, and PCX powder were obtained from Agela Technologies (Tianjin, China). DA was purchased from Sigma (St. Louis, MO, USA), and the purity of DA was determined for 93.4% using a certified calibration solution (101.8 ± 2.1 μg/mL; Lot#20071205), which was obtained from NRC CRM (Halifax, NS, Canada).

### 3.2. Samples Collection and Preparation

Blank urine samples were collected from healthy volunteers and spiked with different amounts of DA. All healthy volunteers signed an informed consent for research purposes prior to the experiment, and all experiments were performed under the approval of the Ethics Committee of China National Center for Food Safety Risk Assessment. A aliquot (100 μL) of sample was accurately measured into a 1.5 mL Eppendorf tube and diluted with 100 μL of 2% ammonium hydroxide. After shaking for 30 s, the mixture was subjected to the PTSPE procedure.

### 3.3. PTSPE Procedure

The PTSPE procedure was carried out with two pipette tips (200 μL and 1000 μL, polypropylene) and an accu-jet^®^ Pro Pipette Controller (Brand, Germany) (Figure 1b). PAX sorbent (9.1 mg) was packed into a pipette tip (200 μL) using two small pieces of degreased cotton capped at both ends of the pipette tip to avoid sorbent loss. Then, the tip of the bigger pipette (1000 μL) was inserted into the smaller pipette tip. The accu-jet^®^ Pro Pipette Controller was used to hold the pipette tips and control the procedure of aspirating and dispensing of the solution. Before extraction, the pipette tip with PAX sorbent was preconditioned with 1.0 mL of methanol and 1.0 mL of water, respectively. After that, the urine sample was aspirated into the conditioned pipette tip, and dispensed back into the same sample tube. These two steps are referred to as one aspirating/dispensing cycle. In this study, at least three repeated aspirating/dispensing cycles were performed for loading the sample extract. Then the tip was washed with 1.0 mL of water and dried under the control by the pipette controller to remove any traces of water. Finally, the target analyte was eluted from the tip with 2 × 185 μL aliquots of 6% formic acid solution (for a total of 370 μL) by two aspirating/dispensing cycles. The collected eluent was ready for LC-HRMS analysis.

### 3.4. Preparation of Standards and Quality Control (QC) Samples

The stock solution of DA was prepared by weighing out approximately 5 mg in 25 mL of methanol. Standard working solutions of DA ranging from 2 μg/L to 100 μg/L were prepared by serial dilution of the stock solutions with water and stored at 4 °C. Seven calibration standards (0.1, 0.2, 0.5, 1, 2, 5, 10 μg/L) were made by adding the working solutions to blank human urine. QC samples including limit of quantification (LOQ), QC low, QC middle and QC high of 0.37, 0.74, 3.7, and 37 μg/L for DA were prepared in the same way.

### 3.5. Chromatographic Conditions

UHPLC analysis was performed on a UHPLC Ultimate 3000 system (Dionex, Sunnyvale, CA, USA) with the column oven temperature maintained at 40 °C, using an HSS T_3_ (2.1 mm × 100 mm, 1.8 μm particle size) analytical column (Waters, Milford, MA, USA). The aqueous solvent (A) consisted of a mixture of 0.1% of formic acid and 4 mM ammonium formate in water and the organic phase (B) was acetonitrile with 0.1% formic acid. The solvent gradient adopted was as follows: 0 min 2% B, 0–2 min 2% B, 2–5 min 50% B, 5–7 min 100% B, 7–7.1 min 2% B, 7.1–10 min, 2% B. The flow rate was set to 200 μL/min with a resulting overall runtime of 10 min. The injection volume was 5 μL.

### 3.6. Mass Spectrometry Conditions

Q-Exactive Mass Spectrometer (Thermo Fisher Scientific, Bremen, Germany) equipped with a heated electrospray ionization (HESI) was operated in the positive (ESI^+^) electrospray ionization modes. The system was controlled by Xcalibur 2.2 software (Thermo Fisher Scientific, Bremen, Germany). The spray voltage was 3.5 kV for the positive mode. The temperature of ion transfer capillary, sheath gas, auxiliary gas, sweep gas, and S-lens RF level were set to 325 °C, 40, 10, 0 (arbitrary units) and 55 V, respectively. The instrument was calibrated in the positive ion mode every three days using the calibration solutions, including caffeine, MRFA, and a mixture of fluorinated phosphazines ultramark 1621, provided by the instrument manufacturer.

The Q-Exactive detector was operated in tMS/MS mode. The tMS/MS scan mode is similar to the MRM scan mode used in the triple quadrupole mass spectrometer. The mass spectrometer acquired a tMS/MS scan at a resolution of 70,000 FWHM with 5.0 e^5^ of Automatic Gain Control (AGC) target (the number of ions to fill *C*-Trap) and 200 ms of maximum ion Injection Time (IT).

### 3.7. Method Validation

The specificity of the method was assessed by comparing the blank urine samples from different individuals with the corresponding spiked urine samples to check for absence of interfering signals of the analyte.

The linearity of the method was checked by examining the calibration curve prepared using seven different concentrations as described above for urine samples. The calibration curve was constructed by plotting the peak area *versus* the spiked concentrations of least square linear regression analysis with a weighting factor of 1/*x*.

The LOD and LOQ were estimated from signals for chromatograms of samples spiked at the lowest concentration validated and corresponded to a signal-to-noise (*S*/*N*) ratio of three and 10, respectively.

The intra-day precision and accuracy were estimated by analyzing five replicates of QC samples at four concentration levels on the same day. In order to determine inter-day precision and accuracy, five replicates of QC samples were analyzed on five different validation days. Precision was calculated as the percentage of relative standard deviations (%RSD) while accuracy was expressed as percentages of deviation (%biases) of the target value which was calculated from the difference between the experimentally determined mean content and nominal concentrations. The criteria for acceptability of the data included within ±15% relative error from the nominal values and a precision of within ±15% RSD.

The analyte recovery for extraction was calculated by comparing the mean area response of the pre-extraction spiked urine samples to that of the post-extraction spiked urine samples at each QC level in six replicates. The matrix effect was expressed as the ion suppression/enhancement on the ionization of analyte and was assessed by comparing the mean area response of post-extraction spiked urine samples with mean area of neat standard solutions (*n* = 3). The effect was considered negligible if values below ±20% were observed. The value of matrix effect less than 80% represented ionization suppression, while more than 120% represented ionization enhancement.

The stability of the analyte in urine samples was determined by repeated analysis (*n* = 3) of QC samples which were stored at −20 °C. The samples were extracted and analyzed after one, two, and four weeks. 

## 4. Conclusions

In the present study, a self-assembly PTSPE method using PAX sorbent combined with high-resolution mass spectrometry was established for the rapid analysis of DA in urine. The merits of the proposed method include as follows: (1) This PTSPE method was conducted without the evaporation and centrifugation steps, and the total time for extraction and cleanup of one sample was short at about 5 min; (2) The cost of the proposed method using PAX adsorbent for one sample was lower (approximately 0.10 US dollars) compared to that of a SPE column (at least 2.0 US dollars for each); and (3) only 100 μL urine sample was required for analysis of DA, while the proposed method combined with UHPLC-HRMS yielded a good detection level (0.12 μg/L), which was sufficiently low to meet the requirements of routine monitoring and exposure assessment. To the best of our knowledge this paper is the first to report the use of a strong anion exchange material (PAX) as a sorbent in the PTSPE procedure for sample preparation of DA in biological fluid.

## Supplementary Materials

The following are available online at www.mdpi.com/2072-6651/8/1/10/s1.
