# Supplementary material for: A Rapid LC-HRMS Method for the Determination of Domoic Acid in Urine Using a Self-Assembly Pipette Tip Solid-Phase Extraction
Source: Toxins (Basel). 2015 Dec 29;8(1):10. doi: 10.3390/toxins8010010 (PMC4728532; doi:10.3390/toxins8010010)
Supplement: Supplementary file 1 [file toxins-08-00010-s001.pdf]

Supplementary Materials: A Rapid LC-HRMS Method for the Determination of Domoic Acid in Urine Using a Self-Assembly Pipette Tip Solid-Phase Extraction

Yiping Zhang, Dawei Chen and Zhuan Hong

Chromatographic Conditions for Analysis of Domoic Acid in Three Different Columns

The aqueous solvent (A) consisted of a mixture of 0.1% of formic acid and 4 mM ammonium formate in water, and the organic phase (B) was acetonitrile with 0.1% formic acid.

HILIC column: 90 min 2% B, 0–2 min 90% B, 2–5 min 50% B, 5–7 min 50% B, 7–7.1 min 90% B, 7.1–10 min, 90% B. The flow rate was set to 300 μL/min with a resulting overall runtime of 10 min. The injection volume was 5 μL.

C_{18} column: 0 min 5% B, 0–2 min 5% B, 2–5 min 50% B, 5–7 min 100% B, 7–7.1 min 5% B, 7.1–10 min, 5% B. The flow rate was set to 300 μL/min with a resulting overall runtime of 10 min. The injection volume was 5 μL.

T_3 column: 0 min 2% B, 0–2 min 2% B, 2–5 min 50% B, 5–7 min 100% B, 7–7.1 min 2% B, 7.1–10 min, 2% B. The flow rate was set to 200 μL/min with a resulting overall runtime of 10 min. The injection volume was 5 μL.

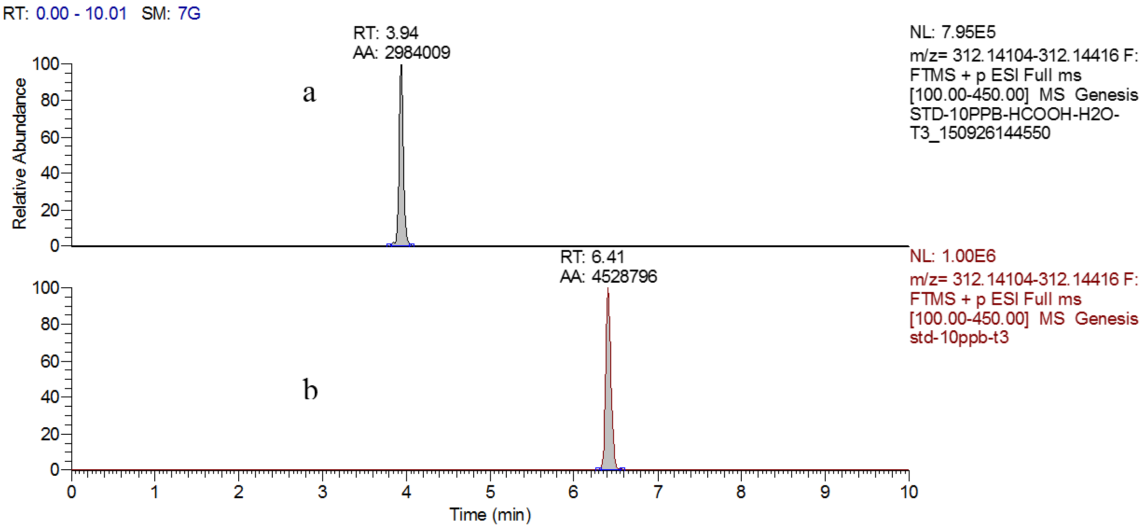

Figure S1. The chromatographic behavior for DA under different linear gradient mode in T3 column with the flow rate of 200 μL/min; (**a**) 0 min 2% B, 0–2 min 2% B, 2–5 min 50% B, 5–7 min 100% B, 7–7.1 min 2% B, 7.1–10 min, 2% B; (**b**) 0 min 10% B, 0–2 min 10% B, 2–5 min 50% B, 5–7 min 100% B, 7–7.1 min 10% B, 7.1–10 min, 10% B.

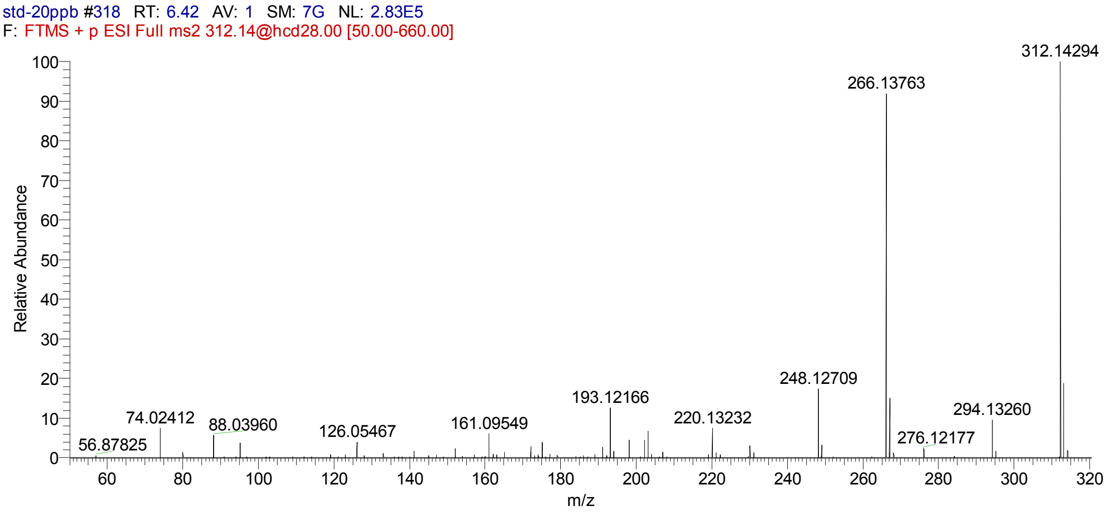

Figure S2. A full scan of all fragmented ions originating from the parent ion (312.14 *m*/*z*) for DA.

Table S1. The extraction conditions for optimization of each parameter by analyzing spiked urine samples containing 1 µg/L DA.

Experiments	The Type of Sorbent	pH	The Amount (mg)	The Number of Aspirating/ Dispensing Cycles	The Concentration of Formic Acid (%)	The Volume of Eluent (µL)
The type of sorbent	C_{18}, C_8, PEP, SCX, SAX, PAX, and PCX	10	10	5	5	5 × 200
pH	PAX	4–10	10	5	5	5 × 200
The amount of sorbent	PAX	10	1–15	5	5	5 × 200
The number of aspirating/ dispensing cycles	PAX	10	10	1–20	5	5 × 200
The concentration of formic acid	PAX	10	10	3	0.5–10	5 × 200
The volume of eluent	PAX	10	10	3	5	1× 200–5 × 200

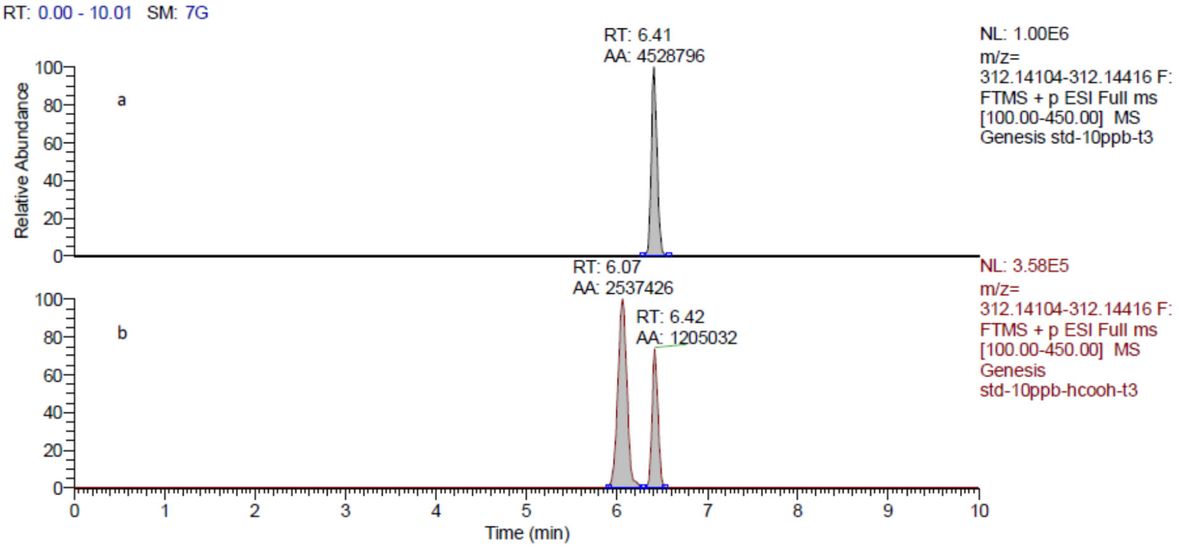

Figure S3. The solvent effects for DA under different solvent in T3 column (**a**) 5% formic acid-water; (**b**) 5% formic acid-acetonitrile.

Table S2. Efficiency of extraction (%) as a function of three independent variables.

Std	Run	Factor *A* (mg)	Factor *B* (%)	Factor *C* (μL)	Response *Y* (%)
18	1	10	5	2 × 200	98
13	2	10	5	2 × 100	89
7	3	7.5	6.5	2 × 250	85
4	4	12.5	6.5	2 × 150	95
20	5	10	5	2 × 200	101
6	6	12.5	3.5	2 × 250	92
12	7	10	8	2 × 200	99
2	8	12.5	3.5	2 × 150	91
10	9	15	5	2 × 200	94
19	10	10	5	2 × 200	97
11	11	10	2	2 × 200	83
15	12	10	5	2 × 200	99
17	13	10	5	2 × 200	97
14	14	10	5	2 × 300	98
5	15	7.5	3.5	2 × 250	92
1	16	7.5	3.5	2 × 150	85
8	17	12.5	6.5	2 × 250	96
16	18	10	5	2 × 200	93
3	19	7.5	6.5	2 × 150	88
9	20	5	5	2 × 200	73

Table S3. ANOVA for response surface quadratic model of DA.

Source	Sum of Squares	*df*	Mean Square	*F*-Value	*P*-Value
Model	766.1591	9	85.12879	6.56904	0.0035
A	272.25	1	272.25	21.00842	0.0010
B	81	1	81	6.250438	0.0314
C	36	1	36	2.777973	0.1265
AB	18	1	18	1.388986	0.2659
AC	0.5	1	0.5	0.038583	0.8482
BC	12.5	1	12.5	0.964574	0.3492
A^2	322.1591	1	322.1591	24.8597	0.0005
B^2	73.05195	1	73.05195	5.63712	0.0390
C^2	29.30195	1	29.30195	2.261111	0.1636
Lack of fit	94.09091	5	18.81818	2.650448	0.1542
Pure error	35.5	5	7.1	-	-
Corrected total	895.75	19	-	-	-
SD	3.60	R^2	0.8553	-	-
CV	3.90	adj-R^2	0.7251	-	-
